# Supplementary figures and images for: Chaperonin containing TCP1 as a marker for identification of circulating tumor cells in blood
Source: PLoS One. 2022 Jun 24;17(6):e0264651. doi: 10.1371/journal.pone.0264651 (PMC9232171; doi:10.1371/journal.pone.0264651)

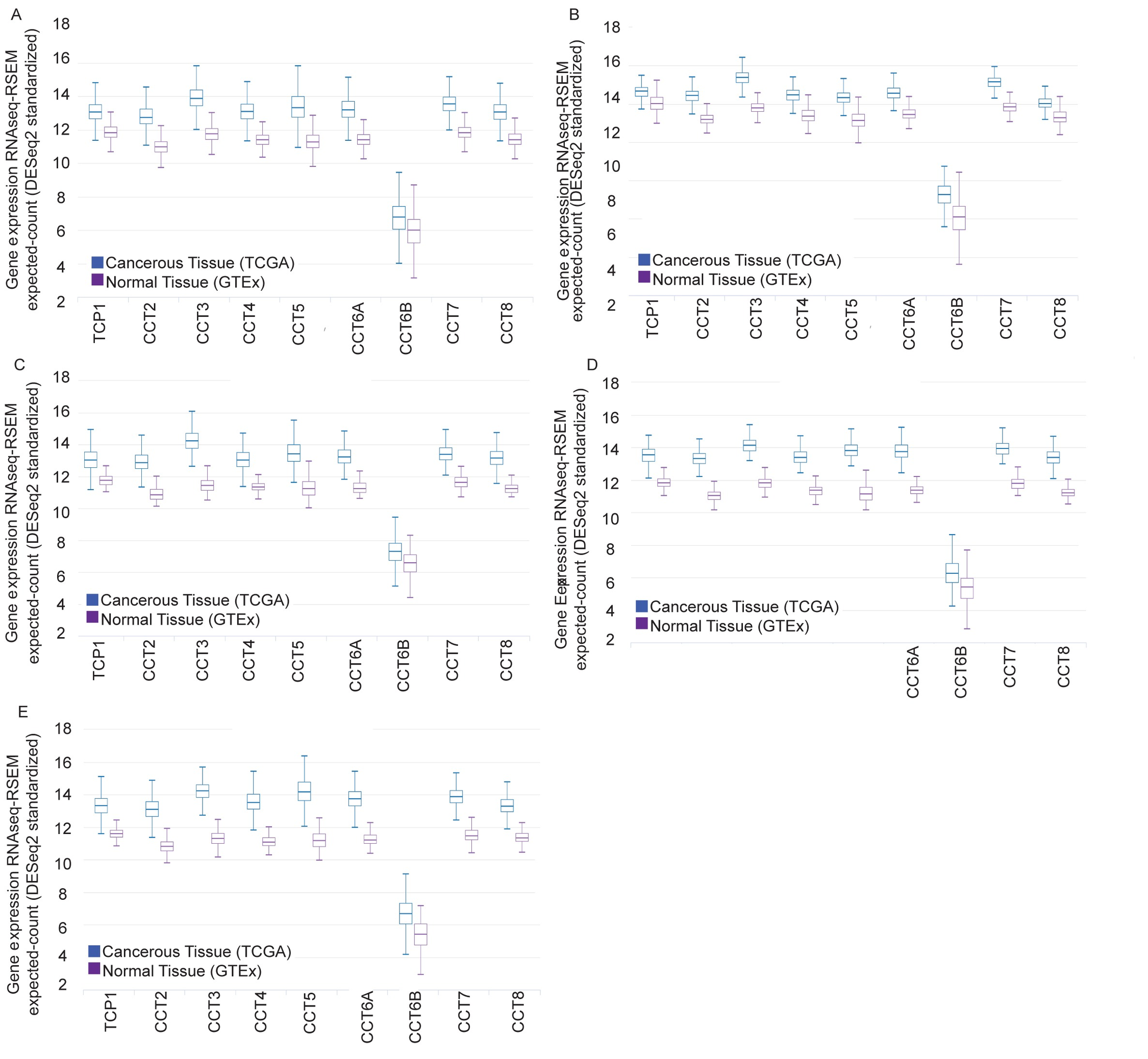

Supplement: S1 Fig — (A) Overall cancer (n = 17,200): the most significant differences were observed in the CCT2 and CCT3 genes, and the least difference was in the CCT6B gene. (B) Brain cancer (n = 1,277): the most significant difference was seen in the CCT2 gene, and the least difference was in the CCT6B gene. (C) Breast cancer (n = 1,660): the most significant differences were seen in the CCT3 and CCT8 genes and the least difference was in the CCT6B gene. (D) Colon cancer (n = 598): the most significant difference was seen in the CCT2 gene, and the least difference was in the CCT6B gene. (E) Lung cancer (n = 1,301): the most significant differences were seen in the CCT2 and CCT3 genes, and the least difference was in the CCT6B gene. p<0.0001 for all samples. (TIF) [file pone.0264651.s001.tif]

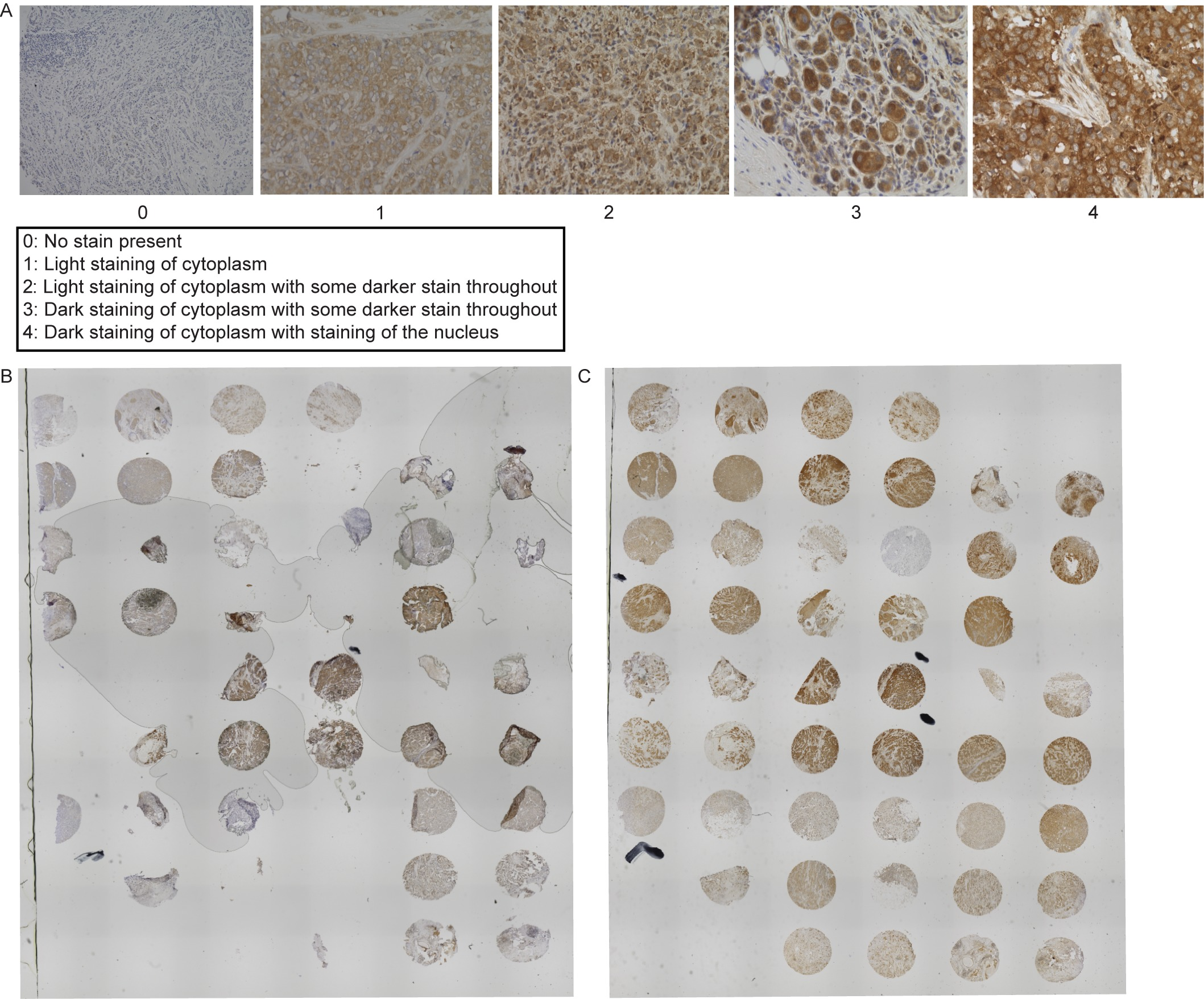

Supplement: S2 Fig — (A) Representative images of breast cancer tissue at each CCT2 stain score 0–4 with an explanation for the stain score scale. Images are from 0: TMABRN801b sample E1, 1: patient 25, 2: patient 08, 3: patient 33, 4: patient 37. (B-C) Breast cancer TMA Core Matrix from Florida Hospital stained for (B) CCT2 and (C) STAT3. (TIF) [file pone.0264651.s002.tif]

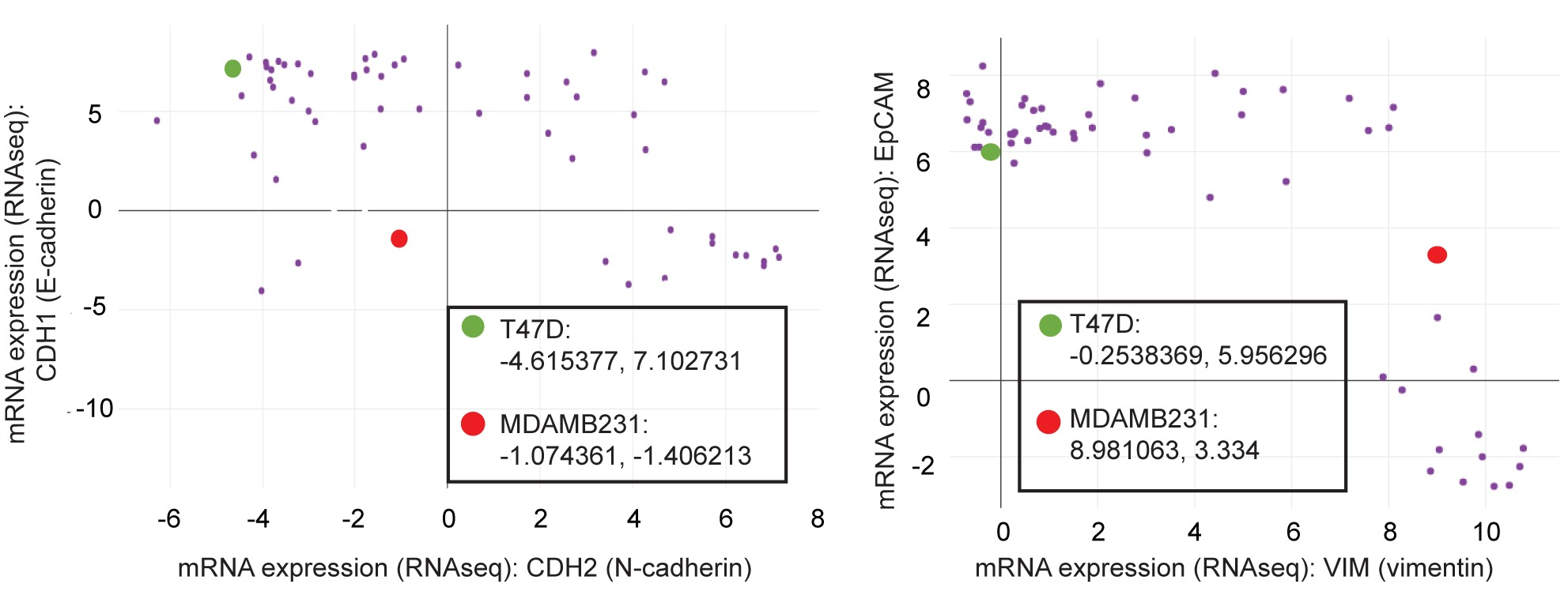

Supplement: S3 Fig — Breast cancer cell lines (purple dots) with a focus on T47D (green dot) and MDA-MB-231 (red dot) cell lines for CDH1 (E-cadherin) vs. CDH2 (N-cadherin), and EpCAM vs VIM (vimentin). (TIF) [file pone.0264651.s003.tif]

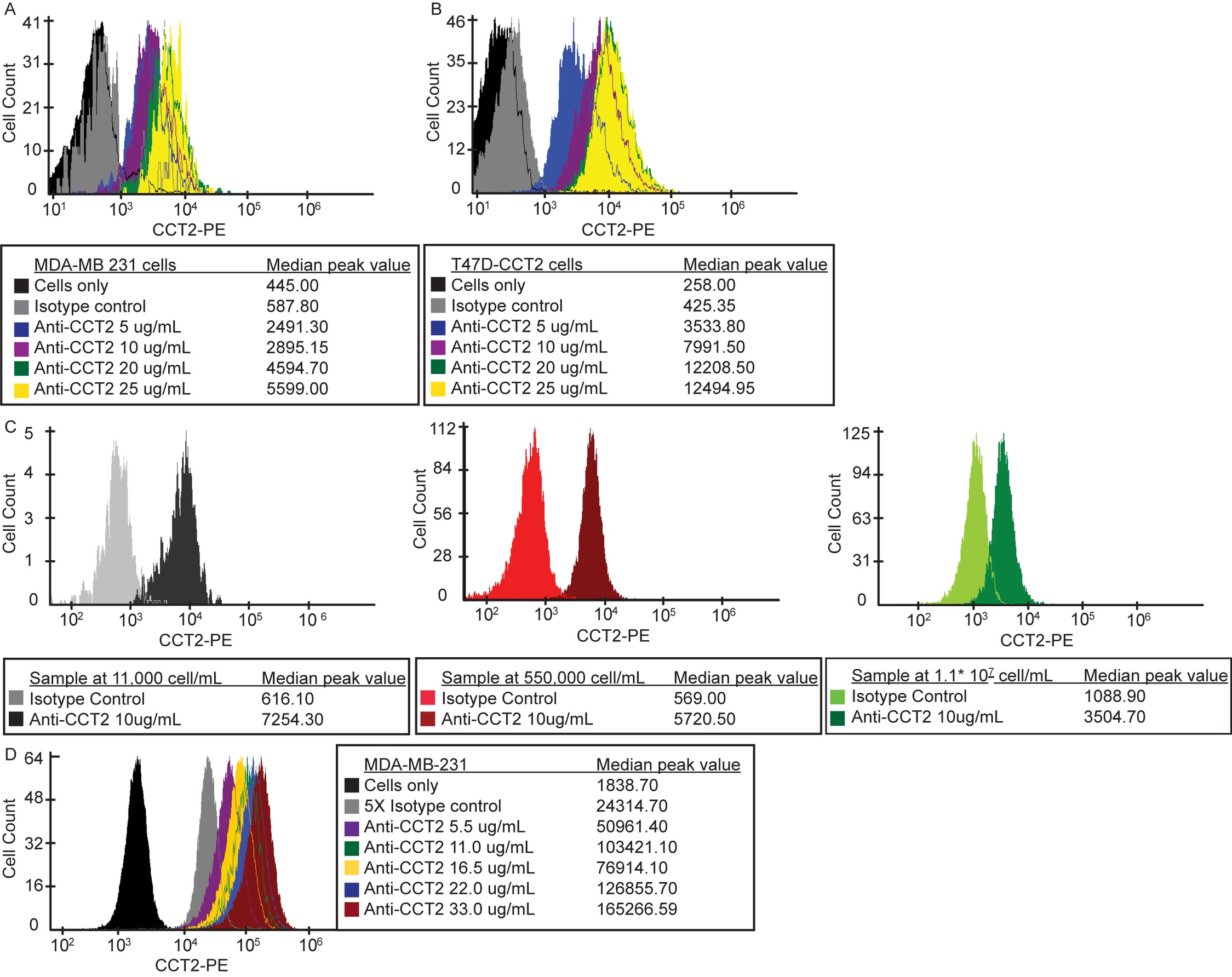

Supplement: S4 Fig — (A-B) Tested anti-CCT2-PE concentrations: 0 μg/ml (black), 5 μg/mL (blue), 10 μg/mL (purple), 20 μg/mL (green), and 25 μg/mL (yellow) in (A) MDA-MB-231 and (B) T47D-CCT2 cell lines with PE-isotype control (grey). (C) Tested various concentrations of MDA-MB-231 cells as indicated, with 10 μg/mL of anti-CCT2-PE (black/dark red/dark green) and PE-isotype control (light grey/red/green). (D) Tested 5X concentration of PE-isotype control (grey) with 70 min incubation in MDA-MD-231 cells with anti-CCT2-PE concentrations: 0 μg/mL (black), 5.5 μg/mL (purple), 11.0 μg/mL (green), 16.5 μg/mL (yellow), 22.0 μg/mL (blue), and 33.0 μg/mL (red). (TIF) [file pone.0264651.s004.tif]

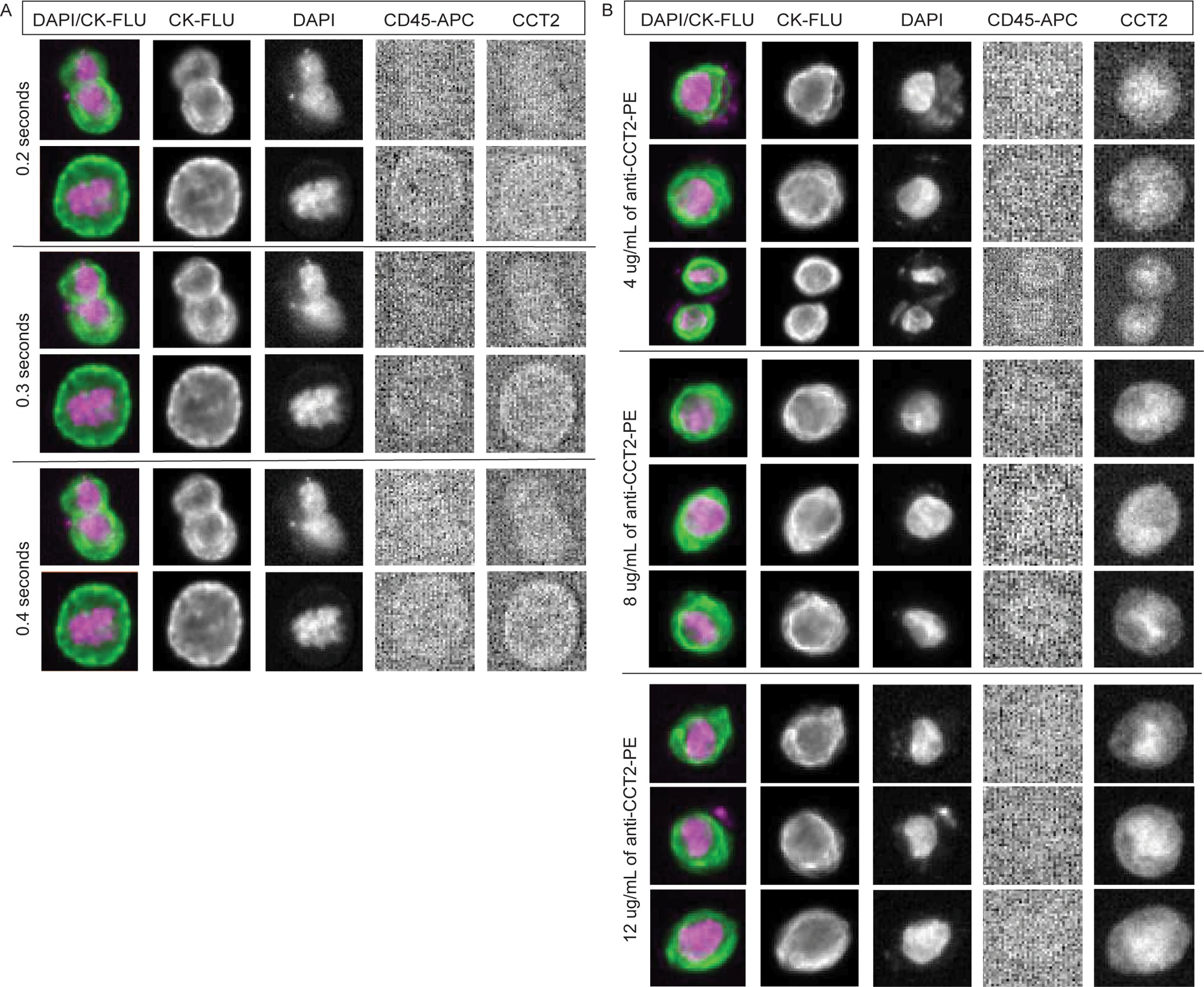

Supplement: S5 Fig — (A) Representative images from CSS Analyzer II of spiked cancer cells stained with 0 μg/mL anti-CCT2-PE at three different exposure times: 0.2, 0.3, and 0.4 sec. (B) Images from CSS Analyzer II of spiked cancer cells at 0.2 seconds exposure stained with three different concentrations of anti-CCT2-PE: 4 μg/mL, 8 μg/mL, and 12 μg/mL as indicated. Column one: overlay of columns two and three. Column two: CK-FLU-FITC signal. Column three: DAPI signal. Column four: CD45-APC signal. Column five: this column was used to stain for anti-CCT2-PE. The experiment was completed in duplicate. (TIF) [file pone.0264651.s005.tif]

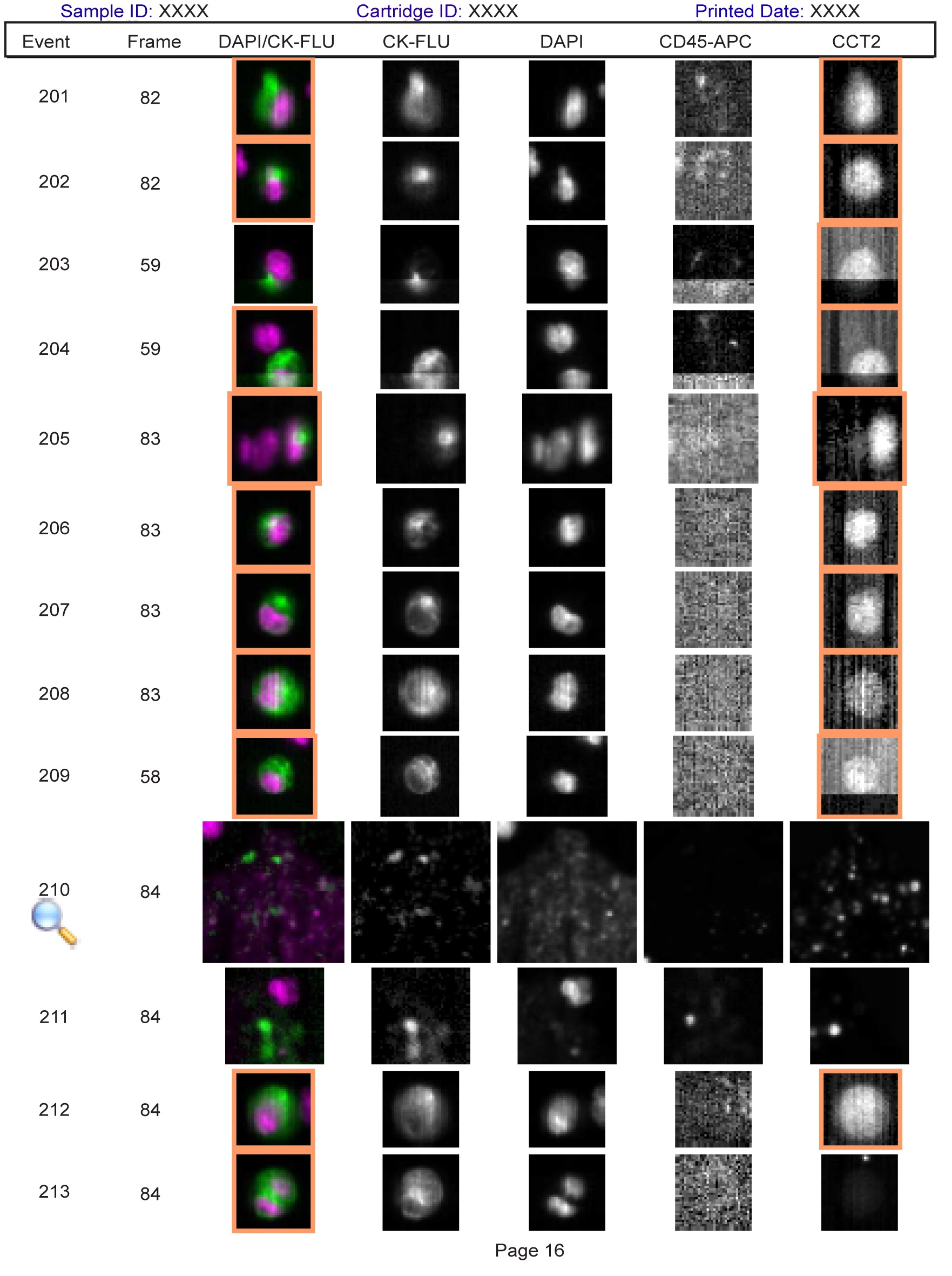

Supplement: S6 Fig — The orange box indicates an event that was selected by the operator as a spiked cancer cell or CCT2 positive cell. Column one: overlay of columns two and three. Column two: CK-FLU-FITC signal. Column three: DAPI signal. Column four: CD45-APC signal. Column five: this column was used to stain for anti-CCT2-PE. This includes representative images from ten experiments. (TIF) [file pone.0264651.s006.tif]

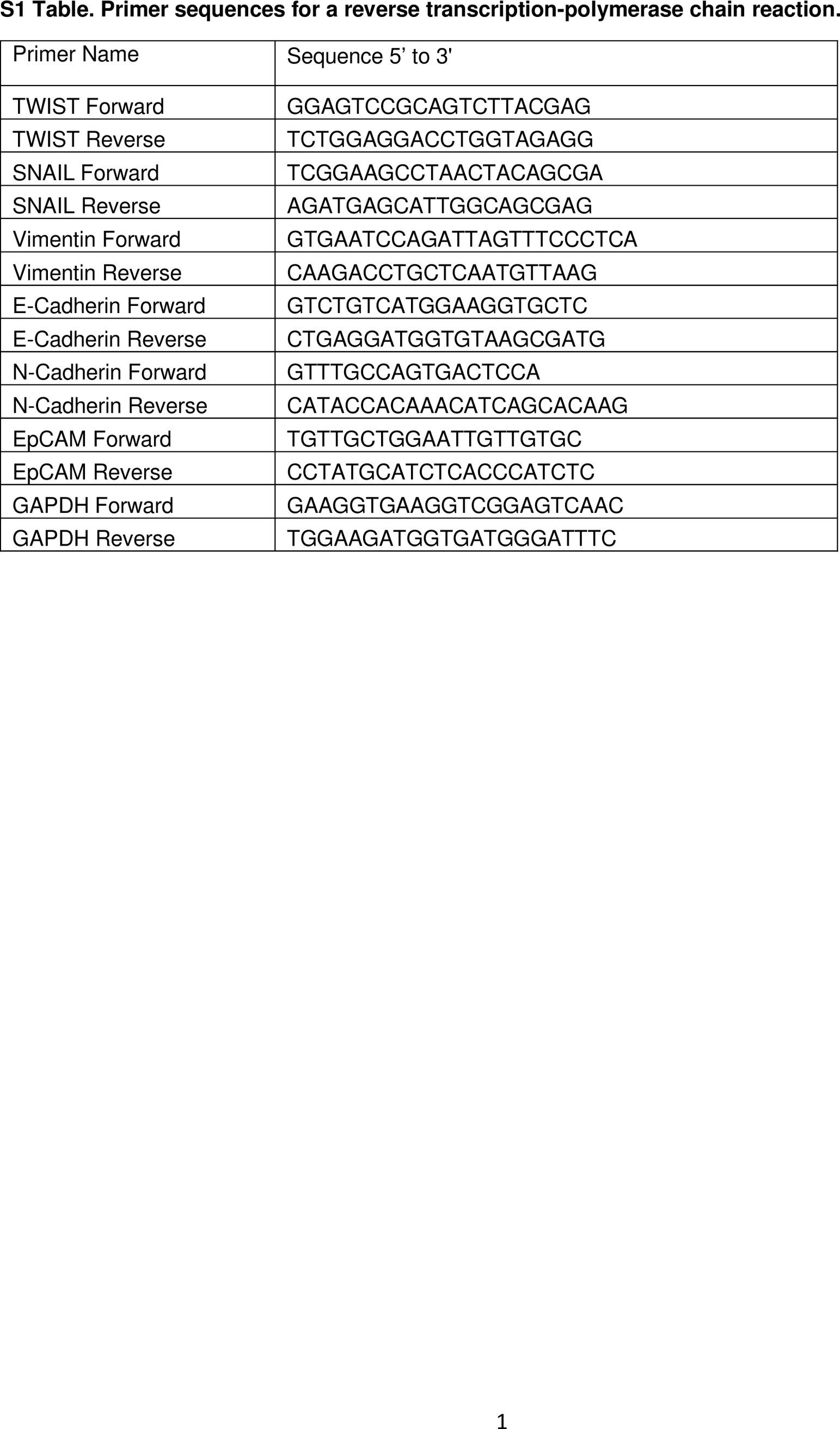

Supplement: S1 Table — (TIF) [file pone.0264651.s007.tif]

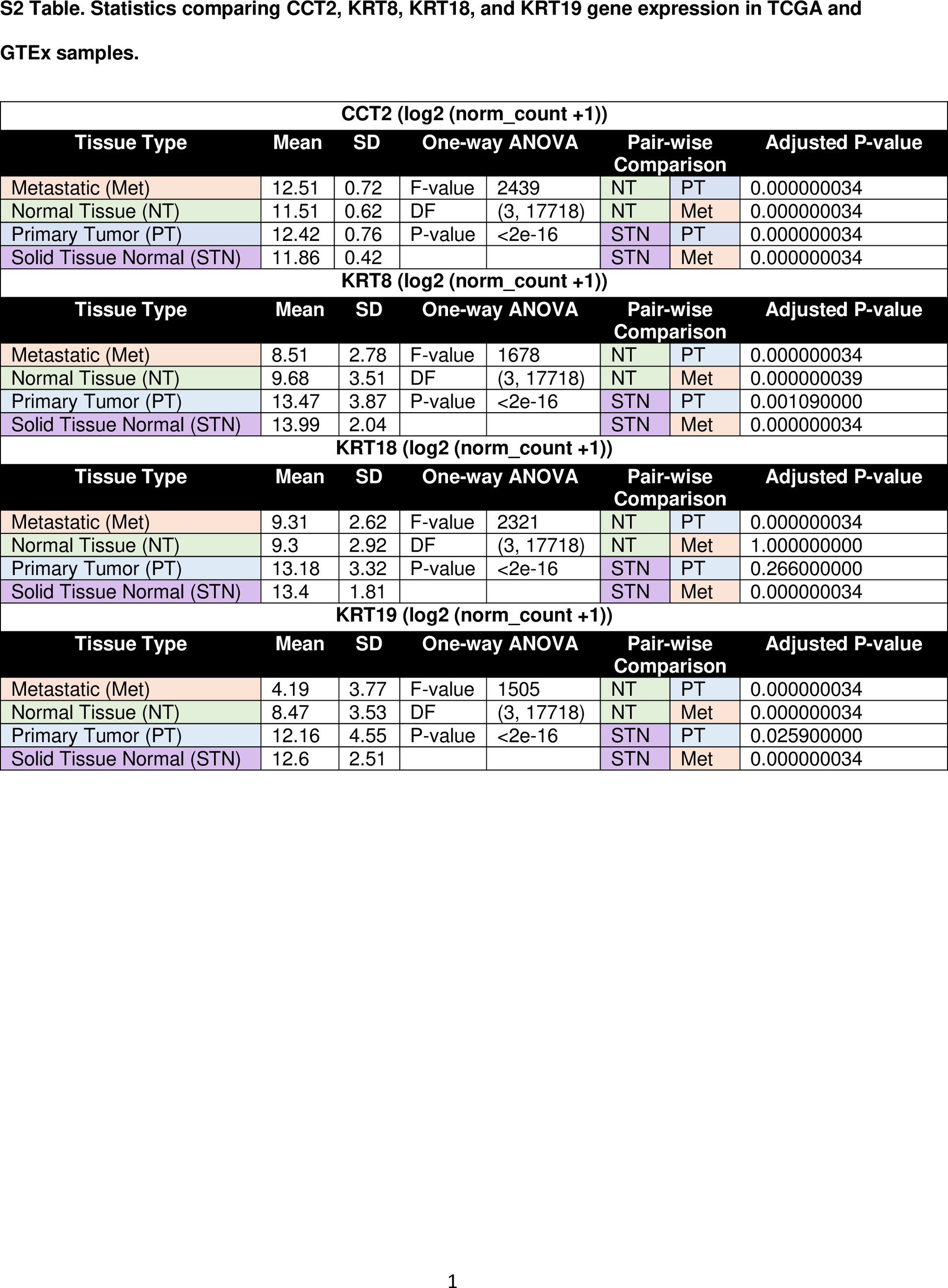

Supplement: S2 Table — (TIF) [file pone.0264651.s008.tif]

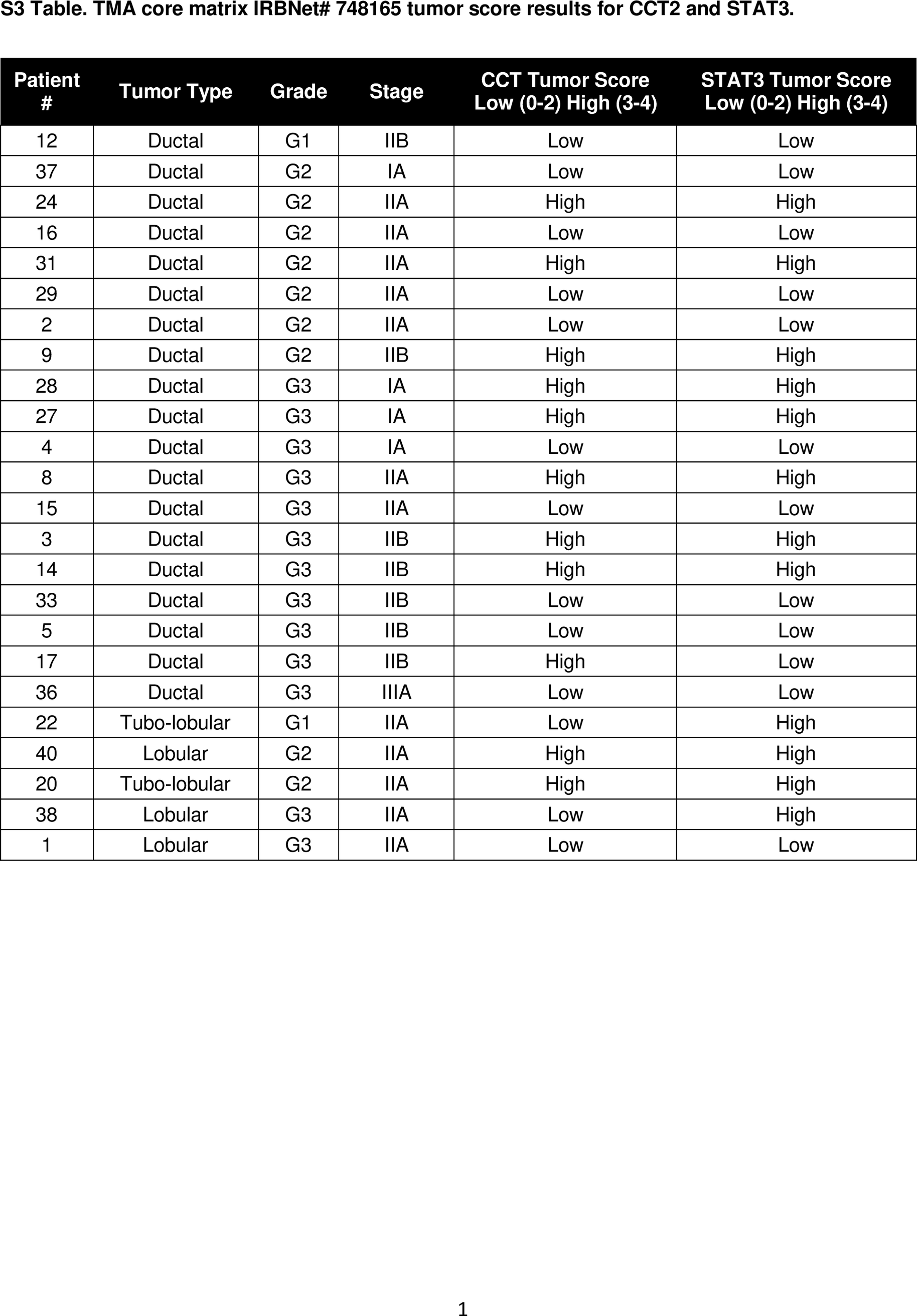

Supplement: S3 Table — (TIF) [file pone.0264651.s009.tif]

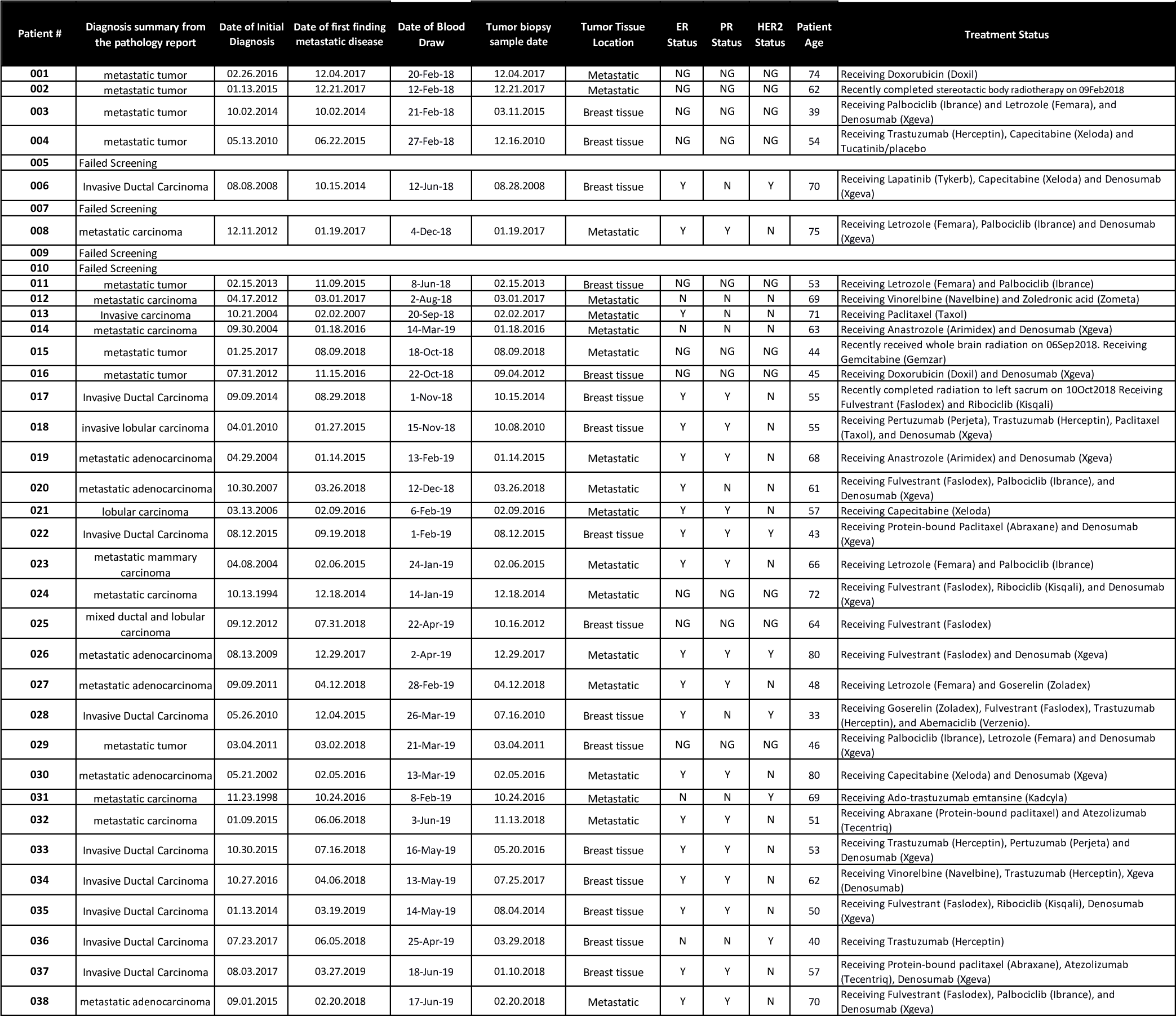

Supplement: S4 Table — NG: information not given, Y: yes, N: no. (TIF) [file pone.0264651.s010.tif]
