# Supplementary material for: Chaperonin containing TCP1 as a marker for identification of circulating tumor cells in blood
Source: PLoS One. 2022 Jun 24;17(6):e0264651. doi: 10.1371/journal.pone.0264651 (PMC9232171; doi:10.1371/journal.pone.0264651)
Supplement: Attachment — Submitted filename: Response to Reviewers .docx [file pone.0264651.s011.docx]

**RESPONSE TO REVIEWERS**

**We thank the reviewers for their constructive comments on our manuscript and appreciate the time and effort to review our manuscript. We’ve addressed each reviewers’ comment below and added additional new data to support the conclusions made. We also carefully edited our manuscript to adhere to publication guidelines.**

Reviewer #1:

The manuscript entitled "Chaperonin Containing TCP1 as an actionable marker for detection of rare cancer cells shed in blood" highlighted that detection of CCT2 could identify tumor cells shed into blood and has application in liquid biopsy approaches, such as detection of CTCs, to enable the use of minimally invasive methods for cancer diagnosis.

The manuscript is really well written and of interest for the readers.

Minor comments:

1. The Authors should provide the expand forms for all acronyms, including gene acronyms, thorugh the text when they first appear.
2. Gene acronyms should be written in italics.

**We thank the reviewer for these comments and all acronyms have been spelled and gene names listed in italics. As example, italics were used for gene names like *CCT2* and *CDH1* and “protein” added when in reference to protein levels.**

3. The Authors should provide high quality figures.

**In the resubmitted manuscripted, high quality figures were separately uploaded.**

**Reviewer #2:**

Main comment:
Authors evaluated the use of intracellular CCT2 staining for visualization of breast cancer cells spiked into blood from healthy donors. "This approach served as a surrogate for cancer patient blood."
If i did not misunderstand, authors did not use the patients blood sample. I would strongly suggest to have used the patients blood samples and the healthy donors as controls not as a surrogate.

**We thank the reviewer for drawing our attention to the use of heathy donors and addressed our reasons for their use below.**

1. Please change the topic of this manuscript with a more specific one as its not clearly informative.

**We appreciate this suggestion and made edits to the title, abstract, and throughout the manuscript to better explain the rationale for study.**

2. the introduction and the method section is too long and difficult to follow. the text should be more into point and straight forward. also some subheadings could help (method section).

**In response to this comment, we shortened the introduction to three paragraphs and the methods section was edited to remove extraneous details and citations to published methods were used where appropriate.**

3. please clarify why the authors did not use the patients blood sample.

**We thank the reviewer for this comment since it may also be a question for readers of the manuscript. Our study focuses on a new biomarker for detection of circulating tumor cells (CTCs). These are rare cells shed into blood from cancers. CTCs are not present in every blood sample taken from a cancer patient. As example, we show in our paper (Table 1) that out of 23 metastatic breast cancer (MBC) patients only 3 patients had had 5 or greater CTCs in one tube of blood. It also took us 2 years to collect these MBC patient samples. Our oncologists can recruit 12-15 MBC patients per year to participate in a study. You can imagine that it would take us many years to optimize a new protocol for detecting CTCs using patient blood if less than 15% of MBC patients have detectable CTCs. Instead, because our goal was to demonstrate that Chaperonin-Containing TCP-1 subunit 2 (CCT2) could be used to detect tumor cells shed in blood, we used the approach of spiking blood from heatlhy donors with known numbers of breast cancer (or lung cancer) cells. Typically, blood from heatlhy individuals will not have any CTCs as we show in Fig. 4A. Since we could control the number of cancer cells added to a tube of blood, this approach let us test the feasibility of using CCT2 to identify rare cancer cells from the abundant non-cancer cells (e.g., leukocytes) found in blood. The following are examples of the many references using similar blood spiking cancer cell methods.**

- **Potential Applications for Circulating Tumor Cells Expressing the Insulin-Like Growth Factor-I Receptor. PMID: 17575225, DOI: 10.1158/1078-0432.CCR-07-0268**
- **Molecular Biomarker Analyses Using Circulating Tumor Cells. PMID: 20838621, PMCID: PMC2935889, DOI: 10.1371/journal.pone.0012517**
- **Technical validation of a new microfluidic device for enrichment of CTCs from large volumes of blood by using buffy coats to mimic diagnostic leukapheresis products. PMID: 33219265, PMCID: PMC7680114, DOI: 10.1038/s41598-020-77227-3**

**However, we did appreciate that the reviewer’s comment was a valid concern. So we opted to commercially purchase blood from patients with small cell lung cancer (SCLC) since such patients typically have more detectable CTCs in blood than MBC patients. Given the time frame to revise our manuscript, we were able to secure blood from four SCLC patients through a commercial vendor. Note that SCLC comprises only about 15% of lung cancers, so collecting such samples from our local patient population would take many months. Using our CCT2-based CTC detection protocol, we confirmed the conclusions made from the cancer cell blood spiking experiments that CCT2 staining for identification of CTCs is feasible using patient blood and that enumeration of more CTCs could be possible, even with cancers like SCLC in which CTC prognostic threshold are not established. This new data has been added to the manuscript in Figure 7. The reviewer can also appreciate that we cannot make statistical conclusions with 4 patient samples and that to achieve this would require months to years of collecting likely 100s of cancer patient blood samples, which is well beyond the scope of our manuscript. The addition of the 4 patient samples does, however, support our conclusions from the spiked blood experiments and demonstrate the feasibility of using CCT2 as marker for CTCs as was our intent.**
